# Efficacy data of halogenated phenazine and quinoline agents and an NH125 analogue to veterinary mycoplasmas

**DOI:** 10.1186/s12917-020-02324-4

**Published:** 2020-04-06

**Authors:** Marissa A. Valentine-King, Katherine Cisneros, Margaret O. James, Robert W. Huigens, Mary B. Brown

**Affiliations:** 1 Department of Environmental and Global Health, College of Public Health and Health Professions, University of Florida, Gainesville, Florida, USA; 2 Department of Medicinal Chemistry, College of Pharmacy, University of Florida, Gainesville, Florida, USA; 3 Department of Infectious Diseases and Immunology, College of Veterinary Medicine, University of Florida, Gainesville, Florida, USA

**Keywords:** Veterinary mycoplasmas, Drug evaluation, Quinoline, NH125 analogue, Phenazine, Nitroxoline

## Abstract

**Background:**

Mycoplasmas primarily cause respiratory or urogenital tract infections impacting avian, bovine, canine, caprine, murine, and reptilian hosts. In animal husbandry, mycoplasmas cause reduced feed-conversion, decreased egg production, arthritis, hypogalactia or agalactia, increased condemnations, culling, and mortality in some cases. Antibiotics reduce transmission and mitigate clinical signs; however, concerning levels of antibiotic resistance in *Mycoplasma gallisepticum* and *M. capricolum* isolates exist. To address these issues, we evaluated the minimum inhibitory concentrations (MICs) of halogenated phenazine and quinoline compounds, an *N-*arylated NH125 analogue, and triclosan against six representative veterinary mycoplasmas via microbroth or agar dilution methods. Thereafter, we evaluated the minimum bactericidal concentration (MBC) of efficacious drugs.

**Results:**

We identified several compounds with MICs ≤25 μM against *M. pulmonis* (*n* = 5), *M. capricolum* (*n* = 4), *M. gallisepticum* (*n* = 3), *M. alligatoris* (n = 3), *M. agassizii* (*n* = 2), and *M. canis* (*n* = 1). An *N-*arylated NH125 analogue, compound 21, served as the most efficacious, having a MIC ≤25 μM against all mycoplasmas tested, followed by two quinolines, nitroxoline (compound 12) and compound 20, which were effective against four and three mycoplasma type strains, respectively. Nitroxoline exhibited bactericidal activity among all susceptible mycoplasmas, and compound 21 exhibited bactericidal activity when the MBC was able to be determined.

**Conclusions:**

These findings highlight a number of promising agents from novel drug classes with potential applications to treat veterinary mycoplasma infections and present the opportunity to evaluate preliminary pharmacokinetic indices using *M. pulmonis* in rodents as an animal model of human infection.

## Background

The Mollicutes, a class of wall-less, fastidious bacteria, cause infections primarily in the respiratory and urogenital tracts in humans, and similarly infect a wide array of animal species including avian, bovine, canine, caprine, murine, and reptilian hosts. Disease negatively impacts aspects of animal husbandry by decreasing feed conversion, egg and milk production, and increasing condemnations and culling [1, 2]. In wildlife, mycoplasmas cause upper respiratory tract (URT) disease in threatened species, including desert and gopher tortoises in the U.S., as well as fulminant disease in the American alligator [3, 4]. Lastly, mycoplasma infection in laboratory animals can skew results and alter immune responses [5]. Use of antibiotics in animals alleviates clinical signs and decreases shedding and hence transmission. However, heavy use of antimicrobials has resulted in increased and sometimes substantial levels of antibiotic resistance in *Mycoplasma gallisepticum* and *M. capricolum* isolates [6–8]. As mycoplasmas lack a cell wall, this further restricts available treatment to those that interfere with either protein synthesis or DNA replication [9]. Further, as mutations that cause resistance against one drug in a class can extend resistance to others within the same class, novel antibiotics are desperately needed to ensure animal and hence, human health [10].

*M. gallisepticum* serves as the most pathogenic and economically impactful mycoplasma to infect poultry [1]. In chickens, it causes a classic triad of pneumonia, tracheitis, and airsacculitis, also termed ‘chronic respiratory disease [9].’ In turkeys, *M. gallisepticum* causes a characteristic ‘infectious sinusitis,’ resulting in severe mucopurulent sinusitis and infraorbital swelling, and airsacculitis that leads to more severe outcomes such as respiratory distress [1]. Illness associated with *M. gallisepticum* infection negatively impacts commercial flocks by causing reduced feed consumption, weight loss, reductions in egg production, mortality, and carcass downgrading or condemnation upon processing [1]. Although primary prevention methods are employed, outbreaks occur and treatment with broad spectrum antibiotics reduces mortality, pathology, clinical signs, egg production losses, and transmission [1].

However, studies have identified concerning and rising minimum inhibitory concentrations (MICs) in *M. gallisepticum* isolates. For example, a 2008 Israeli study detected increases in *M. gallisepticum* MICs to enrofloxacin among isolates from turkeys, and an Israeli study in 2011 found resistance to enrofloxacin, tylosin, and tilmicosin in 72% of *M. gallisepticum* isolates from 2006 and onward [8]. Further, a study in Jordan found rising MICs over time to all macrolide (*n* = 3), quinolone (*n* = 2), and tetracycline (*n* = 3) compounds tested [6]. *M. gallisepticum* isolates collected from myriad of countries between 1986 and 2010 found varying levels of enrofloxacin resistance in isolates from England (33%, *n* = 1), the Netherlands (37.5%, *n* = 3), Israel (46%, *n* = 23), Germany (53.3%, *n* = 8), and Austria (75%, *n* = 3) [11]. This study also highlighted increasing trends in resistance, as 61% of isolates from 2004 and onward displayed enrofloxacin resistance, compared to only 5.8% of isolates collected pre-2004 [11]. To supplement our summary, Table 1 highlights antibiotic resistance prevalence and MIC_50/90_ values extracted from recent studies across diverse, geographical settings. A recent review by Gautier-Bouchardon provides a more comprehensive summary of antibiotic resistance trends among *M. gallisepticum* field isolates [8].

**Table 1:** Antibiotic resistance prevalence and MIC_50/90_ values of *M. gallisepticum* field isolates to select antibiotics

				No. (%) Resistance and MIC_50/90_ values (μg/mL)									
Study	Country	Years	Isolate Total	Enro Res.	Enro MIC_50/90_	Ery Res.	Ery MIC_50/90_	Til. Res.	Til MIC_50/90_	Tylosin Res.	Tylosin MIC_50/90_	ChlTet Res.	ChlTet MIC_50/90_
[12]^a^	Israel	1997–2005	32	7 (22)	na	na	na	12 (38)	na	12 (38)	na	na	na
		2006–2010	18	16 (89)	na	na	na	13 (72)	na	13 (72)	na	na	na
Total			50	23 (46)	0.25/5	na	na	25 (50)	0.1/≥10	25 (50)	0.05/2.5	na	na
[11]^a^	AU	1986–1995	8	0	na	na	na	na	na	na	na	na	na
	US	1996–2008	5	0	na	na	na	na	na	na	na	na	na
	UK	2004–2005	3	1 (33.3)	na	na	na	na	na	na	na	na	na
	GER	2006–2010	15	8 (53.3)	na	na	na	na	na	na	na	na	na
	Austria	2008–2010	4	3 (75)	na	na	na	na	na	na	na	na	na
	NE	1999–2005	8	3 (37.5)	na	na	na	na	na	na	na	na	na
[6]^a,b^	Jordan	2004–2005	22	1 (4.5)^b^	≤ 0.03 / ≤ 0.03	2 (9.1)^b^	≤ 0.03 / 4	2 (9.1)^a^	≤ 0.03 / ≤ 0.03	0^b^	≤ 0.03 / ≤ 0.03	0^c^	1 / 2
		2007–2008	7	5 (71.4)^b^	2 / 8	5 (71.4)^b^	≥64 / ≥64	4 (57.1)^a^	2 / 32	1 (14.3)^b^	0.125 / 4	1 (14.3)^c^	4 / 32
[13]^b^	Egypt	2012–2014	14	na	na	5 (35.7)	4/32	na	na	2 (14.3)	0.25 / 4	na	na
[14]^b,^^c^	SA	2003–2015	10	0	0.25 / 1	na	na	na		6 (60)	10 / 16	2 (20)^c^	4 / 16

^a^Resistance breakpoints to tylosin (≥ 0.63 μg/mL), enrofloxacin and tilmicosin (≥ 1.25 μg/mL), were extracted from Gerchman et al. [12]

^b^Resistance breakpoints to enrofloxacin (≥ 2) and erythromycin (> 4) were extracted from Hannan et al. [15] and the resistance breakpoint to tylosin (≥ 4 μg/mL) was extracted from Beylefeld et al. [14]

^c^Oxytetracycline resistance breakpoint (≥ 16 μg/mL) used for chlortetracycline per AU-Australia; ChlTet-chlortetracycline; Enro-enrofloxacin; Ery-erythromycin; GER-Germany; na-not tested; NE-Netherlands; No-number; Res-resistance; SA-South Africa; Til-tilmicosin; UK-United Kingdom; US-United States

*Mycoplasma capricolum* sub. *capricolum* (*Mcc*), detected primarily in regions that support small ruminant dairy production in Europe, the Mediterranean, North Africa, and sporadically in the U.S., serves as one of the three principal agents that causes contagious agalactia (CA) [16]. *Mcc*, more prevalent in goats than sheep, can cause severe serous or fibrinopurulent arthritis, mastitis, hypogalactia or agalactia, and abortion in adults and death in kids [16]. In countries that depend on goat and sheep products as large dietary sources or exports, economic losses from agalactia, abortions, reduced growth, culling or death due to *Mcc* can have substantial impacts [2]. To avoid economic losses, the industry relies on vaccines and antibiotics. A trivalent, killed vaccine incorporating a *Mcc* antigen exists; however, there is little data available regarding its efficacy [17]. Unlike *M. gallisepticum*, less data on antibiotic resistance in *Mcc* exists. A recent study evaluating antibiotic resistance in 32 *Mcc* field isolates from the Canary Islands, mainland Spain, and Italy found notable levels of resistance to erythromycin (100%), norfloxacin (77.4%), spectinomycin (64.5%), clindamycin (48.4%), and tylosin (19.4%) [7]. In contrast, a study performed in Jordan from 2002 to 2003 found no resistance and strikingly lower MIC_50/90_ values to erythromycin, tylosin, and enrofloxacin [18]. Table 2 highlights additional findings from these two studies.

**Table 2:** Antibiotic resistance prevalence and MIC_50/90_ values of *M. capricolum* subsp. *capricolum*

				(%) Resistance and MIC_50/90_ values (μg/mL)									
Study	Country	Years	Isolate Total	Enro Res.	Enro MIC_50/90_	Ery Res.	Ery MIC_50/90_	Til Res.	Til MIC_50/90_	Tylosin Res.	Tylosin MIC_50/90_	Clind Res.	Clind MIC_50/90_
[18]^a^	Jordan	2002–2003	8	0	0.25 / 0.25	0	< 0.03 / < 0.03	na	na	0	< 0.03 / < 0.03	na	na
[7]^a^	Italy, Spain	2005–2016	32	2 (6.5)	0.2 / 0.4	32 (100)	> 12.8 / > 12.8	4 (12.9)	0.025 / > 12.8	6 (19.4)	0.1 / > 12.8	15 (48.4)	0.2 / > 12.8

^a^Resistance breakpoints for enrofloxacin (≥ 2 μg/mL), erythromycin (≥ 1 μg/mL), tilmicosin (≥ 32 μg/mL), tylosin (≥ 4 μg/mL) and clindamycin (≥ 0.5 μg/mL) used from Tatay-Dualde [7]. Clind-clindamycin; Enro-enrofloxacin; Ery-erythromycin; Res-resistance; Til-tilmicosin

*M. canis*, designated as an opportunistic pathogen, primarily causes urogenital tract disease but has been associated with granulomatous or necrotizing meningoencephalitis in canines [9, 19, 20]. Likely through canine-cattle interactions, *M. canis* has been identified in cattle from Canada and northern Europe, and was detected in 13 pneumatic calf outbreaks in Britain during the mid- to late-1990s [17]. *M. canis* served as the sole agent detected in five outbreaks, wherein three outbreaks reported calf mortality [17, 21]. Recently, *M. canis* was isolated from wound tissues of a German woman after a dog bite [22]. No studies have investigated antibiotic resistance in *M. canis* isolated from either dogs or cattle.

Mycoplasmas that infect reptilians include, but are not limited to *M. alligatoris*, a virulent pathogen of alligators and caimans, and *M. agassizii*, a pathogen that causes URT disease in tortoises. *M. alligatoris* was initially discovered as the causative agent of an outbreak among captive alligators that caused interstitial pneumonia, fibrinous pericarditis, arthritis, and 80% herd mortality [4]. Seroprevalence studies across Florida detected 60% seropositivity among 20 sites tested, and 5.4% seropositivity overall among 32 samples [23]. In-vitro studies found *M. alligatoris* isolates had low MICs (< 1 mg/L) to doxycycline, enrofloxacin, tilmicosin, and tylosin, but higher MICs against erythromycin (32–128 mg/L), chloramphenicol (8–16 mg/L), and clindamycin (1–8 mg/L) [24].

In the 1980s, an URT infection coupled with other factors coincided with substantial declines in the desert tortoise (*Gopherus agassizii*) population in the Mojave Desert in California, with similar disease occurring in wild and captive gopher tortoises (*G. polyphemus*) in Florida [25]. Isolation, sequencing, and experimental infection studies led to identification of *M. agassizii* as a unique mycoplasma species that causes dyspnea, nasal discharge, rhinitis, and conjunctivitis in desert and gopher tortoises [3, 26]. Although enrofloxacin has been used to treat infected tortoises, it does not completely eliminate the organism [3].

*M. pulmonis*, which causes pathology in both the respiratory and urogenital tracts, as well as otitis media, conjunctivitis, and arthritis, infects both captive and wild rodents [9, 27]. *M. pulmonis* infection in laboratory rats and mice, in concert with its effects on the immune system, can confound research studies, especially as subclinical infections can escape detection [5]. *M. pulmonis* genital and respiratory mycoplasmosis prevalence in laboratory rats has been reported in up to 40% and in nearly 100% of conventionally-maintained animals, respectively [27]. Antibiotics are employed during rederivation to prevent vertical transmission following embryo transfer [5, 28]. Urogenital and respiratory infection models in rodents have been established for *M. pulmonis*; therefore novel antibiotics found effective against *M. pulmonis* could undergo pharmacokinetic and pharmacodynamic (PK/PD) analysis using these infection models to determine preliminary parameters [27, 29].

**Fig. 1:**
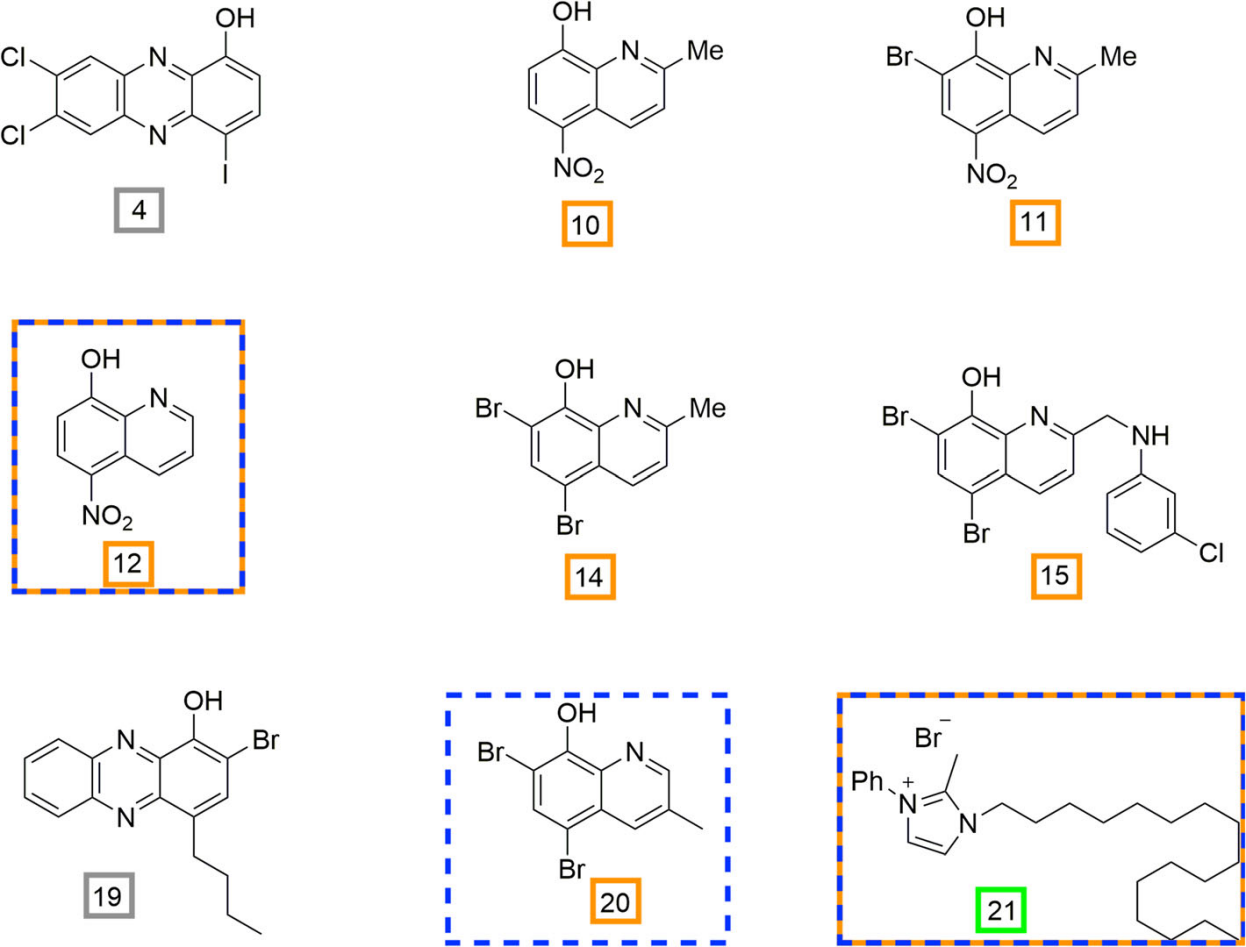
Compounds synthesized by the Huigens Lab. Halogenated phenazine and quinoline compounds, and an *N-*arylated NH125 analogue have gray, orange, and green boxes around compound numbers, designating each class, respectively. Compounds with an orange and blue dotted box surrounding their structure represent those that had the most frequent MICs ≤25 μM against the veterinary mycoplasmas and demonstrated bactericidal activity against four mycoplasmas. A blue, dotted box represents the third most efficacious compound

Herein, we evaluated the MICs and minimum bactericidal concentrations (MBCs) of a collection of seven halogenated phenazine and quinoline compounds, an *N-*arylated NH125 analogue, nitroxoline, and triclosan against six veterinary *Mycoplasma* spp. type strains (Fig. 1). The library of halogenated phenazine and quinoline compounds and the NH125 analogue were created by using a previously efficacious compound as a base structure and modifying different chemical groups at targeted sites to produce a library of more potent phenazine, quinoline, and NH125 analogues [30–32]. Nitroxoline, a compound approved for treating urinary tract infections outside of the U.S., was added to the testing as it has a similar structure to the compounds in the library. Triclosan was included as an agent known to be effective against several microorganisms [33, 34]. In a previous study, we tested this library of novel agents against clinical isolates of *Ureaplasma* spp. and *M. hominis,* as well as against human mycoplasma type strains [35]. We found a number of compounds with efficacious MICs against several human mycoplasmas that have displayed elevated resistance patterns in recent years [35]. With antibiotic resistance increasing in both human and animal mycoplasmas and limited therapeutic options available for mycoplasma treatment, new classes of antibiotics are needed in both human and veterinary medicine. Further, as well-established murine models of mycoplasma respiratory and urogenital tract infections exist, compounds effective against *M. pulmonis* could serve as a stepping stone for establishing important PK/PD parameters for furthering these compounds along the translational spectrum.

## Results

### MIC results (Table 3)

Overall, an *N-*arylated NH125 analogue (compound 21), nitroxoline (compound 12), and a quinoline (compound 20), proved most effective against the veterinary mycoplasmas. Compound 21 had MICs ≤25 μM (11.6 mg/L) to all type strains (*n* = 6), and had a median MIC of 15.7 μM (7.3 mg/L) (95% CI: 12.5–25 μM) against these organisms. Nitroxoline had MICs ≤25 μM (4.8 mg/L) against four type strains: *M. alligatoris, M. capricolum*, *M. gallisepticum*, and *M. pulmonis*, with a median MIC of 12.5 μM (2.4 mg/L) (95% CI: 6.25–25 μM) against these four organisms. Compound 20 had MICs ≤25 μM (7.9 mg/L) against three type strains, *M. alligatoris, M. capricolum,* and *M. gallisepticum,* but had a slightly higher median MIC of 18.8 μM (5.93 mg/L) (95% CI: 12.5–25 μM) against these organisms. Compound 10, a quinoline, and compounds 11 and 14, halogenated quinolines, were solely effective against *M. pulmonis* and each had a MIC of 12.5 μM. Compound 15, a halogenated quinoline and compound 19, a halogenated phenazine, had MICs of 25 μM against *M. agassizii* and *M. capricolum*, respectively. Compound 4 served as the only test compound that did not have a MIC ≤25 μM against any of the type strains.

**Table 3:** MIC results of test agents against veterinary mycoplasma type strains

	MIC (μM) for the following compounds									QC Drug μM, (μg/mL)	No. (%) AMCs w/ MICs ≤25 μM^f^
Organism	4^f^	10	11^f^	12	14	15	19	20	21	Enrofloxacin	
*M. alligatoris*	> 12.5	> 25	> 12.5	25	> 25	> 25	> 25	18.8^a^	25	0.17, (0.06)	3 (33.3)
*M. agassizii*	> 12.5	> 25	> 12.5	> 25	> 25	25	> 25	> 25	18.8^a^	0.04–0.17, (0.02–0.06)	2 (22.2)
*M. canis*	> 12.5^b^	> 25	> 12.5^b^	> 25	> 25	> 25	> 25^b^	> 25^d^	12.5^a^	0.17–0.35, (0.06–0.13)	1 (11.1)
*M. capricolum*	> 12.5	> 25	> 12.5	12.5	> 25	> 25	25	12.5	25	0.4–0.7, (0.13–0.25)	4 (44.4)
*M. gallisepticum*	> 12.5	> 25	> 12.5	6.25^a^	> 25	> 25	> 25	18.8^a^	12.5	0.04, (0.016)^e^	3 (33.3)
*M. pulmonis*^*c*^	> 12.5	12.5	12.5	12.5^a^	12.5	> 25	> 25	> 25	12.5^a^	0.17–0.35, (0.06–0.13)	5 (55.5)
No. (%) AMCs with MICs ≤25 μM^f^	0	1(16.7)	1 (16.7)	4 (66.7)	1 (16.7)	1 (16.7)	1 (16.7)	3 (50)	6 (100)		

^a^Median MIC value from multiple, independent tests

^b^CFU for organism: 1.6 × 10^5^ against this test compound

^c^CFU/mL range or organism: 4.3 × 10^4^–4.4 × 10^5^, CCU/mL: 10^4^–10^6^

^d^Drug MIC confirmed via agar dilution

^e^Used either enrofloxacin or tylosin tartrate for QC drug

^f^Compounds 4 and 11 tested up to 12.5 μM

QC-quality control; AMC-antimicrobial compounds

The organisms most susceptible to the test compounds were *M. pulmonis*, *M. capricolum*, and *M. gallisepticum*, wherein five (55%), four (44%) and three (33%) compounds had MICs ≤25 μM against each, respectively. Among the compounds that registered MICs ≤25 μM against *M. pulmonis* and *M. gallisepticum*, the median MIC for those compounds was 12.5 μM against each; for the four found effective against *M. capricolum*, the median MIC of those compounds was 18.8 μM. Although *M. canis* and *M. agassizii* had fewer compounds that displayed MICs ≤25 μM against them, compound 21 had a low, median MIC of 12.5 μM (5.8 mg/L) against *M. canis* and a median MIC of 18.8 μM (8.7 mg/L) against *M. agassizii*. Triclosan was most effective against reptilian mycoplasmas (*M. alligatoris*, MIC: 120 μM and *M. agassizii*, MIC: 60 μM) and an avian mycoplasma (*M. gallisepticum*, MIC: 120 μM). Triclosan had MICs > 120 μM against the mammalian mycoplasma type strains. The raw MIC data are available in Supplemental File 1.

### MBC results (Table 4)

In terms of the MBC assays, many of the compounds had MICs on the higher end of the doses tested. Therefore, in some cases, the MBC was undetermined as one requires information on growth at 4X the MIC to determine if the drug demonstrates bacteriostatic activity. However, as some drugs had MBCs at the MIC or 2X the MIC level, we were able to ascertain bactericidal activity in those cases. This was the case for nitroxoline and compound 21. Nitroxoline exhibited bactericidal activity against four type strains including *M. alligatoris*, *M. capricolum*, *M. gallisepticum*, and *M. pulmonis*. Among those type strains, nitroxoline had the lowest MBCs against *M. pulmonis* (mean MBC: 17.5 μM; 3.3 mg/L) and *M. gallisepticum* (MBCs: 12.5, 25 μM; 2.4, 4.8 mg/L). Compound 21 demonstrated bactericidal activity against all mycoplasmas that underwent MBC testing (*M. agassizii, M. canis, M. gallisepticum, and M. pulmonis*) in all but two cases (*M. alligatoris, M. capricolum*), wherein the activity was undeterminable. For the remaining test compounds (10, 11, 14, 15, 19, 20), their MBC was undeterminable. Overall, nitroxoline and compound 21 exhibited bactericidal activity against the majority of type strains. The raw MBC data are available in Supplemental File 1.

**Table 4:** MBC data for test agents against veterinary mycoplasma type strains

Compound, organism	MIC (μM)	MBC (μM)^a^	MBC Classification
Compound 10			
*M. pulmonis*	12.5	> 25	Undetermined
Compound 11			
*M. pulmonis*	12.5	> 12.5	Undetermined
Compound 12			
*M. alligatoris*	25	25	Bactericidal
*M. capricolum*	12.5	25	Bactericidal
*M. gallisepticum*	6.25, 25	12.5, 25, respectively	Bactericidal
*M. pulmonis*	12.5	17.5^b^	Bactericidal
Compound 14			
*M. pulmonis*	12.5	> 25	Undetermined
Compound 15			
*M. agassizii*	25	> 25	Undetermined
Compound 19			
*M. capricolum*	25	> 25	Undetermined
Compound 20			
*M. capricolum*	12.5	> 25	Undetermined
*M. gallisepticum*	12.5	> 25	Undetermined
Compound 21			
*M. alligatoris*	25	> 50	Undetermined
*M. agassizii*	25	50	Bactericidal
*M. canis*	12.5, 25	2 X MIC	Bactericidal
*M. capricolum*	25	> 25	Undetermined
*M. gallisepticum*	12.5	25, 50	Bactericidal
*M. pulmonis*	12.5, 25	2 X MIC	Bactericidal

^a^MBC expressed as a factor of MIC when variable MICs obtained during MBC testing

^b^Average MBC from assay conducted five times

## Discussion

Veterinary mycoplasmas inflict substantial fiscal burdens in the poultry, dairy, and beef industries and are often refractory to treatment [1, 2, 6, 36]. Rising antibiotic resistance, mutations that confer resistance to multiple drugs within a single class, mycoplasmas, inherent resistance to major drug classes, and animals with persistent carrier status necessitate the identification of new drug classes [6, 7, 9, 10, 16, 37]. To address these issues, we tested a combination of halogenated phenazines and quinolines, an NH125 analogue, and triclosan against six veterinary mycoplasmas to facilitate identification of new treatment modalities.

We evaluated MICs using methods derived from a standardized, Clinical Laboratory Standards Institute (CLSI) protocol for evaluating resistance among human mycoplasmas [38]. As antibiotic resistance continues to emerge and new drugs need evaluation, a standardized method should be adopted for the purposes of ensuring validity and comparability across studies. Validated, established guidelines can also reduce time spent determining quality control limits and optimizing procedures which will enhance knowledge dissemination and facilitate drug evaluation against mycoplasmas that infect animals.

Overall, we identified compounds in the quinoline and phenazine families and an *N-*arylated NH125 analogue that exhibited MICs ≤25 μM against a diverse group of veterinary mycoplasmas. A previous study found evidence to support that NH125 analogues, such as compound 21, have a mechanism of action that involves rapid bacterial membrane destruction [39]. As mycoplasmas lack a cell wall, leaving its bacterial membrane vulnerable, we posited that NH125 analogues would demonstrate efficacy and bactericidal activity against mycoplasmas. We found evidence for the former hypothesis, as compound 21 had MICs ≤25 μM against all six mycoplasma type strains in this study. Our results reflect a similar trend in compound 21 efficacy against mycoplasmas, as a recent study found that compound 21 displayed low MICs against *M. pneumoniae*, *M. genitalium* (MICs: 3.13 μM), and 72 *Ureaplasma* spp. clinical isolates (MIC_90_: 12.5 μM) [35]. In this study, we found some support for the latter hypothesis, as compound 21 demonstrated bactericidal activity in all scenarios wherein one could determine the MBC, which included bactericidal activity against *M. agassizii*, *M. canis, M. gallisepticum, and M. pulmonis* type strains.

Nitroxoline (compound 12) and compound 20 served as the second and third most effective compounds against the veterinary mycoplasmas, having a MIC ≤25 μM against four and three type strains, respectively. In particular, both had low MICs against *M. capricolum* and *M. gallisepticum*—two mycoplasmas that have had significant and rising levels of antibiotic resistance in recent years, respectively. Further, nitroxoline demonstrated bactericidal activity in all veterinary mycoplasmas tested. This serves as a property which could reduce mycoplasma carrier status among herds or flocks following infection and treatment, when stress could decrease immune clearance of the pathogen.

The collection of halogenated phenazine and quinoline compounds tested in this study originated by probing pyocyanin, a compound produced within the natural setting through bacterial competition. Pyocyanin, a phenazine compound produced by *Pseudomonas aeruginosa* and the presumed compound credited with outcompeting *Staphylococcus aureus* in the context of cystic fibrosis lung infections, served as the base structure used to initiate this exploration [40]. Halogenated phenazine analogue libraries were created by substituting and testing the impact of different chemical moieties at key positions along the pyocyanin cyclic structure. Through scaffold hopping, similar quinoline structures were synthesized that possessed key structural features such as a 1-hydroxy atom positioned adjacently on the second aromatic ring. Later experiments revealed that such positioning created a five-membered chelate, responsible for starving bacterial biofilms by binding with divalent metal cations [41]. Nitroxoline’s mechanism of action also involves divalent, metal ion chelation [42]. Previous studies indicated reduced nitroxoline efficacy in the presence of Mg^2+^ and Mn^2+^ coupled with spectrophotometric absorbance shifts indicating formation of drug-ion complexes for which stability of Mn^2+^ and Mg^2+^ superseded that of Ca^2+^ [42].

In our study, we identified that *M. pulmonis* appeared more susceptible to the halogenated quinoline compounds compared to other veterinary mycoplasmas. Few studies have examined the impact of iron chelation in mycoplasmas. However, one study found that incubating the chelating agent, 2,2′ -dipyridyl for 12 h with *M. pulmonis* at 1 mg/mL versus 0.1 mg/mL decreased the CFU by over 97%, compared to a 50% CFU reduction seen in *M. gallisepticum* [43]. Only after 30 h, treatment with 2,2′ -dipyridyl resulted in a 95% decrease in *M. gallisepticum* CFU [43]. Thus, based on that data, it appears that *M. pulmonis* might have less resilience in dealing with iron sequestration, which could explain why more halogenated quinolines had an impact on *M. pulmonis* compared to *M. gallisepticum.*

For *M. pulmonis*, compounds 10, 11, 12 and 14 also had efficacious MICs compared to sister quinolines 15 and 20. In previous studies evaluating halogenated quinoline libraries against MRSA and MRSE, compounds 15 and 20 demonstrated 1.5 and 4-fold higher MICs to MRSA, and compound 20 had a 6-fold higher MIC to MRSE compared to compound 14 [30, 31]. Thus, it appears this might be reflective of potency seen against other gram-positive organisms, albeit compound 20 had more frequent, efficacious MICs overall against the veterinary mycoplasmas.

It makes sense that compounds 10, 11, 12, and 14 had efficacious MICs against *M. pulmonis* as a cluster, since they have very similar structural motifs. For *M. pulmonis*, it appears that the quinolines proved effective (had MICs at 12.5 μM) when a nitrite was present at the 5-position, regardless of additional methyl or halogenated groups. However, substituting the nitrite with a bromine coupled with addition of a bromine at the 7-position proved effective only when a methyl group was stationed at the 2-position. Interestingly, previous studies evaluating halogenated quinoline against MRSE also identified the 2-position as a key component of the quinoline scaffold for enhanced activity [44]. However, it appears that compound 15, bearing a chlorinated phenol group attached via a nitrogen group, did not show effectiveness. The reason for this is unknown. Further, nitroxoline had more efficacious MICs compared to similar analogues; thus, additional methyl or halogenated groups on the scaffold did not seem to enhance antimicrobial against veterinary mycoplasmas in general.

Compounds that had efficacious MICs against *M. pulmonis* (10, 12, 14, and 21) also had previously efficacious MICs against human mycoplasmas [35]. As *M. pulmonis* infection models have been established in rats for both urogenital and respiratory tract disease [27, 29], one could use these established models to evaluate important PK/PD parameters to determine a compound’s preliminary, therapeutic index. This would contribute to important pre-clinical information to advance knowledge of these compounds to prepare them for clinical studies.

In terms of preliminary safety testing, in-vitro work showed that compounds 14 and 20 produced scant hemolysis at doses of 200 μM (≤ 1%), but compound 15 caused hemolysis in 18.8% of red blood cells at 200 μM [30, 31]. Thus far, the majority of halogenated phenazine compounds have showed no cytotoxicity against HeLa cells at concentrations of 100 μM [41]. However, the *N-*arylated NH125 analogue demonstrated potent hemolysis activity against human red blood cells [32]. Therefore, NH125 analogues may have applications as disinfectants or antiseptics.

As the halogenated phenazine and quinoline compounds contain a hydroxyl group, they are potential substrates for biotransformation via glucuronidation and sulfonation, the same pathways used by triclosan. The glucuronide and sulfate metabolites would likely be inactive and terminate their biological activity. Preliminary studies have shown that although both glucuronide and sulfate metabolites can be formed in human liver microsomes, these compounds are relatively poor substrates that are slowly metabolized [45].

We added further information on the efficacy of triclosan against veterinary mycoplasmas. Herein, we found that triclosan had a MIC of 60 μM (17.4 mg/L) against *M. alligatoris* and MICs of 120 μM (34.7 mg/L) against *M. gallisepticum* and *M. agassizii*. One previous study evaluated triclosan’s efficacy against two distinct *M. gallisepticum* type strains (PG31 and BG44T) and reported similar MICs (32 mg/L; 110 μM) to what we found in the *M. gallisepticum* S6 type strain [46].

Although our work and that of others have shown triclosan to be an effective antibacterial, it is a somewhat controversial chemical. The FDA banned the use of triclosan in soaps and body washes sold to the general public in the U.S. effective September 2017 and banned its use in medical settings effective December 2018. This was because concerns were raised about triclosan’s environmental persistence, the toxicity of triclosan’s breakdown products, and the endocrine-disrupting activities of triclosan itself [47, 48]. However, the FDA permitted triclosan’s continued use in plaque-reducing toothpaste, and it is not banned world-wide.

One issue that plagues husbandry includes subclinical persistence of mycoplasmas following treatment, which can lead to relapse or inadvertent introduction to naïve flocks or herds during transhumance [16, 37]. Further, relapse of *Mcc* has been reported to range between 10 and 30% in herds [16]. Some postulate that biofilms may give rise to carrier status and cause relapse [49]. Thus, antibiofilm activity of antimicrobials against mycoplasmas could serve as an effective property. Several of the compounds found to have lower MICs against these veterinary mycoplasmas (12, 14, 15, 20, and 21) also reported effective eradication activities against MRSA and MRSE biofilms in prior studies [30, 32, 50]. Studies have identified that *M. gallisepticum*, *M. pulmonis* and, to a lesser extent, *Mcc* form biofilms [49, 51, 52]. Thus, future directions include studying the biofilm eradicating properties of these compounds in mycoplasmas, which may have implications for animal and human health.

## Conclusion

Overall, we found a number of compounds belonging to three novel antimicrobial classes that had activity against a group of diverse mycoplasmas that infect food and fiber, companion, reptilian as well as laboratory animals. In determining bactericidal or bacteriostatic activity, we found that nitroxoline possessed bactericidal activity against all veterinary mycoplasmas tested, while an NH125 analogue had bactericidal activity against *M. agassizii*, *M. canis, M. gallisepticum,* and *M. pulmonis*. This property may serve as a useful characteristic to limit mycoplasma carrier status, which contributes to ongoing mycoplasma transmission and subsequent health and agricultural losses. Further, as the majority of compounds showed activity against *M. pulmonis*, which serves as an existing animal model to study both respiratory and urogenital mycoplasmas in humans, this could serve as a starting point to calculate essential, pre-clinical compound data.

## Methods

### Mycoplasma type strains

For this study, we evaluated the test compounds against the following six veterinary mycoplasma type strains: *M. agassizii* ATCC 700616, *M. alligatoris* ATCC 700619, *M. canis* PG14 (ATCC 19525), *Mycoplasma capricolum* sub. *capricolum* ATCC 27343, *M. gallisepticum* S6 (ATCC 15302), and *M. pulmonis* × 1048. For stock culture growth and MIC/MBC testing of *M. agassizii*, *M. alligatoris*, and *M. canis* type strains, we used our standard, laboratory prepared SP4 medium and agar supplemented with glucose with a pH range between 7.6–7.8. For *M. capricolum*, *M. gallisepticum*, and *M. pulmonis* culture and MIC/MBC testing, we used our standardized, laboratory prepared Frey’s medium and agar, supplemented with glucose at a pH between 7.6–7.8.

### Antimicrobial compounds

For quality control purposes, we used enrofloxacin and tylosin tartrate sourced from Sigma Aldrich (St. Louis, MO, USA). Stock solutions of quality control agents were dissolved and diluted according to CLSI standards, and drug purity was accounted for during the dilution process [53]. We stored stock solutions in 1 mL aliquots at − 20 °C for up to 3 months. MICs obtained from quality control drugs that were within a four-fold dilution range were considered acceptable for quality control purposes. The test compounds and triclosan were provided at either 10 mM or 1 mM concentrations in DMSO and were stored at room temperature, protected from light. Drugs were diluted in broth on the day of testing and tested within 6 months of receipt.

### MIC determination

We followed a previously validated, microbroth or agar dilution method to evaluate MICs as previously described [35, 38]. For the microbroth dilution assay, we used sterile, 96-well plates wherein each row contained an antimicrobial agent in doubling dilutions from 25 μM to 3.13 μM for each type strain, in duplicate, unless otherwise noted in Table 3. Duplicate growth control, drug control, solvent control, and medium controls were set up for each drug and organism tested. A 1:10 dilution of DMSO served as the solvent control. Plates were inoculated with 175 μL of organism between 10^4^ and 10^5^ CFU/mL, unless otherwise indicated, which was pre-incubated in broth for either 1 hour for *M. alligatoris* or for 2 hours for all other mycoplasmas tested. Plates were sealed with sterile acetate sealers in ambient air and incubated at 37 **°**C for all mycoplasma type strains except for *M. alligatoris* and *M. agassizii*, which were incubated at 30 **°**C. When the growth control displayed a distinct color change, the MIC was read and interpreted as the lowest concentration of drug that inhibited any color change. We confirmed the organism CFU and CCU on the date of testing and reported results when an organism’s CFU fell outside of this range in two cases. MIC readings were confirmed with a second, independent test. In some cases, multiple MICs were obtained from the initial MIC testing and from conducting the MBC assays. Under these circumstances, the median MIC and the corresponding 95% confidence interval was reported for each drug/organism combination.

As compound 12 (nitroxoline), altered broth color due to its yellow hue at concentrations of 12.5 μM and higher, drug control wells at drug concentrations ≥12.5 μM were placed adjacent to the drug and organism wells, so as to represent the baseline color for that drug concentration in broth. The MIC was interpreted as the lowest drug concentration with no visible color change compared to the corresponding control well.

In the event that a compound and organism combination did not show a distinct color change in broth, which occurred when testing compound 20 and triclosan against *M. canis*, we confirmed the MIC using a validated agar dilution method to evaluate drug MIC. Briefly, the method consisted of incorporating 600 μL of antibiotic within 5.4 mL of molten agar by adding the appropriate volume of stock antibiotic to yield concentrations spanning from 25 to 3.13 μM for each drug. We created a solvent and growth control plate by mixing 5.4 mL of molten agar with 600 μL of a 1:10 DMSO solution and with 600 μL of filter-sterilized, double-distilled water, respectively. Following a two-hour pre-incubation period, we added three separate 10 μL drops of organism at 10^3^, 10^4^ and 10^5^ CFU/mL concentrations onto each agar plate. Using the organism dilution between 10^4^ and 10^5^ CFU/mL, the MIC for each drug was read as the lowest antibiotic concentration that inhibited colony formation when the growth control plate exhibited colonies.

### MBC determination

We evaluated the MBCs of compounds that had MICs ≤25 μM by adapting a previously published method [54]. The MBC assay called for transferring 30 μL aliquots directly from the MIC microtiter plate at 1, 2 and 4 times the MIC drug concentration into culture tubes with 2.97 mL of fresh broth immediately following MIC interpretation. For positive and negative controls, we transferred 30 μL from the growth control and 30 μL from the medium control into separate tubes with 2.97 mL of fresh broth. Following inoculation, all tubes were incubated at 37 °C (or at 30 °C for reptilian mycoplasmas) in ambient air for 14 and 10 days for *M. agassizii* and *M. gallisepticum*, respectively, and for 7 days for all other veterinary mycoplasmas. Compounds were considered bactericidal if the lowest concentration that did not show growth was within one to four times the predetermined MIC level following incubation. We replicated the MBC assay for compounds that registered an MBC value considered bactericidal.

## Supplementary information

**Additional file 1.**

## Data Availability

The datasets used and analyzed during the current study are available from the corresponding author on reasonable request. All mycoplasma type strains used in testing are available through the American Type Culture Collection (https://www.atcc.org), with the exception of *M. pulmonis* × 1048, which can be requested through the Mycoplasma Culture Collection (http://iom-online.org/node/28).

## Abbreviations

CA: Contagious agalactia
CLSI: Clinical Laboratory Standards Institute
MBC: Minimum bactericidal concentration
Mcc: *Mycoplasma capricolum* sub. *capricolum*
MIC: Minimum inhibitory concentration
PD: Pharmacodynamic
PK: Pharmacokinetic
URT: Upper respiratory tract
